# Campbell Title Registrations to Date ‐ January 2020

**DOI:** 10.1002/cl2.1078

**Published:** 2020-03-11

Details of new titles for systematic reviews or evidence and gap maps that have been accepted by the Editor of a Campbell Coordinating Group are published in each issue of the journal. If you would like to receive a copy of the approved title registration form, please send an email to the Managing Editor of the relevant Coordinating Group.

## 1 EDUCATION

*The effects of inclusion on academic achievement, socioemotional development and wellbeing of children with special educational needs*
Nina Thorup Dalgaard, Anja Bondebjerg, Bjørn Christian Arleth Viinholt, Trine Filges
17 December 2019
*The effect of different class sizes on students’ academic achievement, social emotional learning, and wellbeing in special education: A systematic review*
Anja Bondebjerg, Nina Thorup Dalgaard, Trine Filges, Morten Kjær Thomsen, Bjørn Christian Arleth Viinholt
18 December 2019

## 2 INTERNATIONAL DEVELOPMENT

*Participant racial and ethnic diversity in trials testing non‐diet approaches to weight management in US adults: A systematic review*
Cristina Bergman, Purnima Madhivanan
6 December 2019
*The effect of whole‐grain dietary intake on non‐communicable diseases: A systematic review*
Wasim Iqbal, Gavin Stewart, Abigail Smith, Chris Seal
15 December 2019
*Interventions to increase youth employment: An evidence and gap map*
Howard White, Caroline Otike, Thomas Katairo, Sussana Puerto, Drew Gardiner, Alison Annet Kinengyere, John Eyres, Ashrita Saran, Ekwaro Obuku, Robert Apunyo
1 December 2019

## 3 SOCIAL WELFARE

*The support needs of families living with parental mental illness: A qualitative systematic review*
Emma Loudon, Gavin Davidson, Kathryn Higgins
18 December 2019

